# Giant Nevus Lipomatosus Cutaneous Superficialis in the Vulva: Finding the Best Treatment

**DOI:** 10.7759/cureus.50388

**Published:** 2023-12-12

**Authors:** Jesus Ivan Martinez-Ortega, Felipe de Jesus Perez-Hernandez, Jose Alfredo Soto Ortiz, Jorge Carlos Guillermo-Herrera

**Affiliations:** 1 Department of Dermatology, Dermatological Institute of Jalisco, “Dr. José Barba Rubio”, Zapopan, MEX; 2 Department of Internal Medicine, High Specialty Regional Hospital of the Yucatan Peninsula, Merida, MEX; 3 Department of Dermatology, University of Guadalajara, Guadalajara, MEX; 4 Department of Research, High Specialty Regional Hospital of the Yucatan Peninsula, Merida, MEX

**Keywords:** treatment choices, giant tumor, primary cutaneous, vulva neoplasm, nevus lipomatosus superficialis

## Abstract

This report presents a rare case of a giant Nevus Lipomatosus Cutaneous Superficialis (NLCS) on the vulva of a 38-year-old female. The patient underwent excisional surgery and electrodesiccation for complete lesion removal. Recurrence following CO_2_ laser treatment was observed. The study highlights the challenges in managing large NLCS lesions in challenging locations and emphasizes the importance of combining surgical excision and electrodesiccation for successful treatment. Further research and reported cases are needed to enhance our understanding of this rare condition and guide optimal treatment strategies.

## Introduction

The Nevus lipomatosus cutaneous superficialis (NLCS) is a rare benign hamartoma of the skin with no association with any malignancy predisposition. There are no known predisposing factors, but there are some theories on the pathology such as degenerative changes in the dermal connective tissue leading to adipose metaplasia, the alterations in adipose tissue development due to genetic or environmental factors, and the growth of fat cells from the walls of dermal blood vessels. NLCS is characterized by the presence of mature adipocytes within the dermis, without continuity with the subcutaneous adipose tissue. On very rare occasions the lesions can be giant; the lesions are mainly located in the lower back, buttocks, and thighs. To the best of our knowledge, this is the first report of a giant NLCS on the vulva [1].

## Case presentation

**Figure 1:**
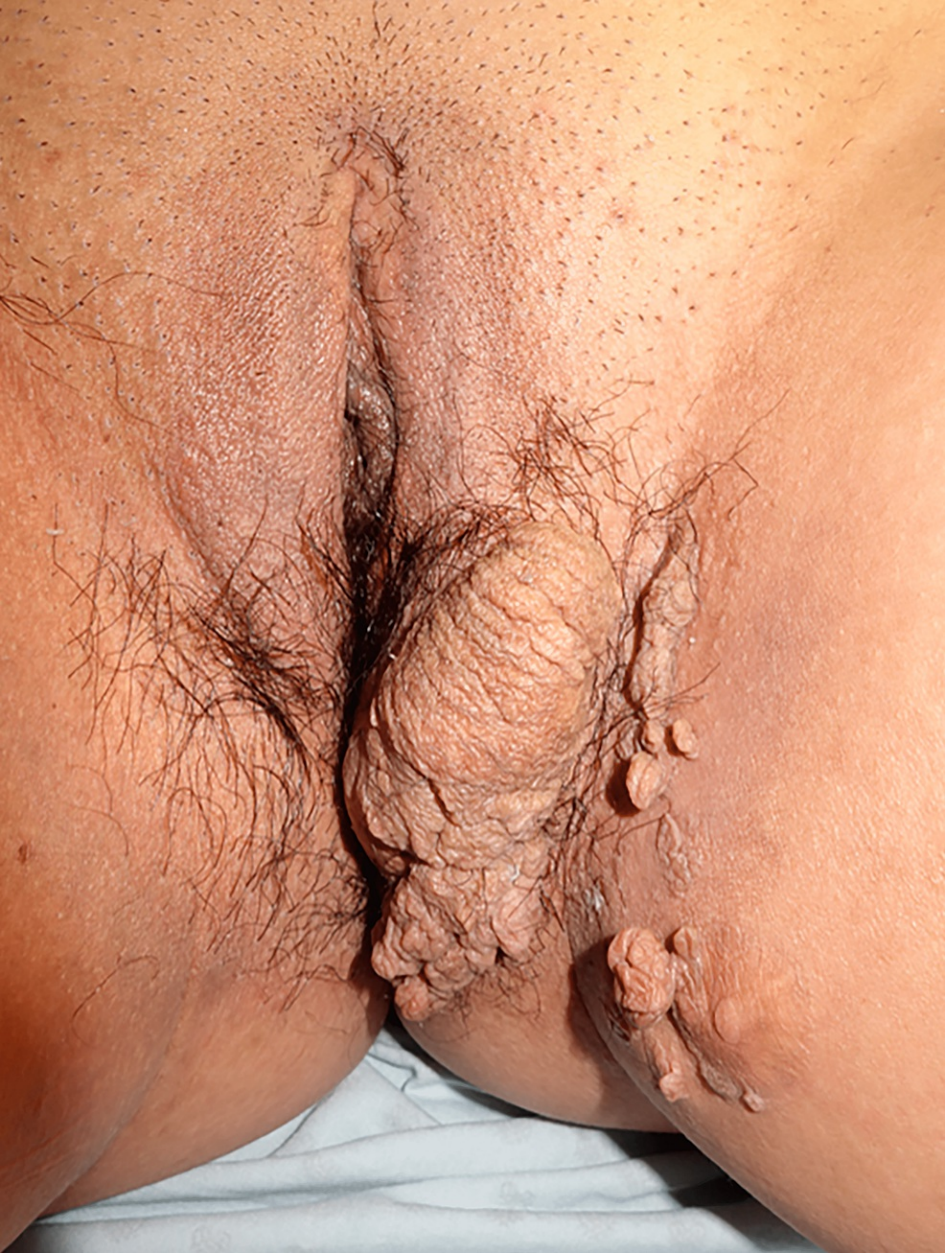
Clinical Image Multiple soft, skin-colored nodules that coalesce to form a large voluminous tumor measuring 20 cm x 10 cm. The tumor exhibits a distinctive cerebriform surface and is primarily located on the vulva. Additionally, smaller nodules can be observed on the medial aspect of the left thigh.

A 38-year-old female presented to the dermatology clinic with multiple soft, skin-colored nodules forming a voluminous tumor (20 cm x 10 cm) with a cerebriform surface on the vulva and medial aspect of the left thigh. She reported 30 years of evolution with gradual growth of the lesion, it was referred asymptomatic, but she complained of aesthetic discomfort. There was no other relevant medical history (Figure 1).

**Figure 2:**
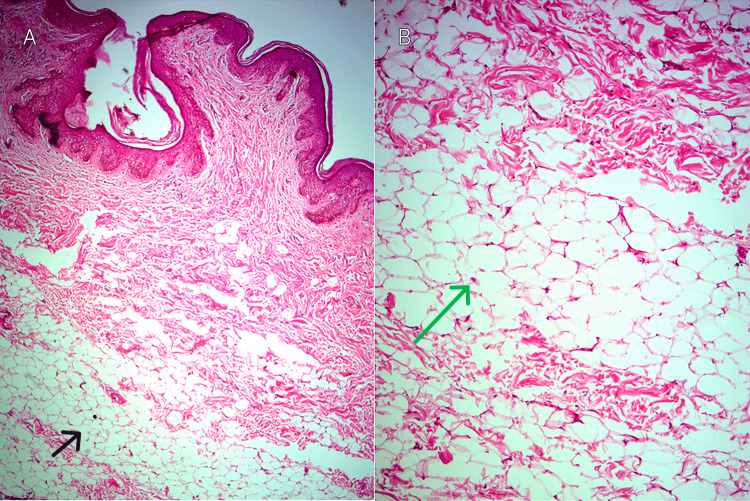
Histopathological image Histopathological image stained with Hematoxylin and Eosin displaying normal epidermis and mature adipocytes distributed within the collagen bundles in both the reticular (black arrow) and papillary dermis (green arrow). (A) Image at 10x magnification, while (B) Closer view at 40x magnification.

The patient had previously undergone complete treatment with CO_2_ laser for the condition 20 years ago but experienced a recurrence of the lesions in the months following the treatment. Histopathologic examination revealed mature adipocytes in the papillary and reticular dermis, leading to the diagnosis of NLCS (Figures 2A, 2B).

**Figure 3:**
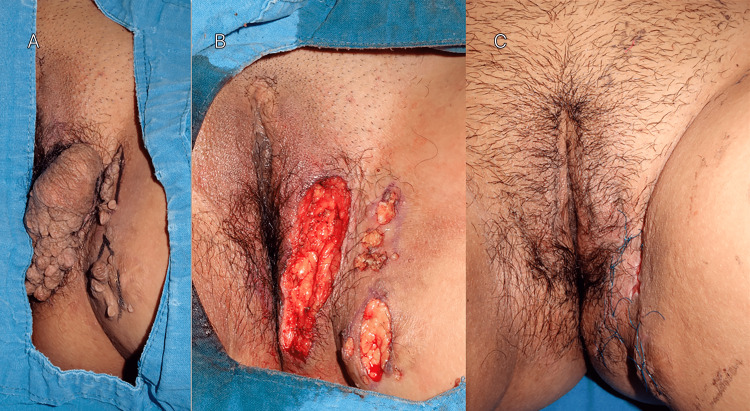
Clinical evolution The image captures the initial presentation of the lesions on the vulva and medial aspect of the left thigh (A), the process of treatment (B), and the post-treatment appearance (C).

The lesions were completely removed by excisional surgery and electrodesiccation. The patient expressed satisfaction with the cosmetic outcome and remained free of recurrence (Figures 3A-3C).

## Discussion

NLCS is typically classified into two types: the classic type, usually occurs at birth or during the first three decades of life and consists of multiple, soft skin-colored or yellowish papules or nodules, covered with smooth or wrinkled skin, sometimes with a cerebriform appearance, and the solitary type is expressed as a skin-colored, single nodule or papule without predilection for a specific age group or a typical location [1].

There is a lack of literature on this subject, with only a few reported cases. The largest reported size of NLCS was 106 cm x 30 cm [2]. Goucha et al. conducted a series of eight NLCS cases, with all but one treated through surgery [3]. In addition, Alotaibi et al. [4] reported a series of five NLCS cases, all of the cases reported in both series most areas affected the lumbar region, the pelvic girdle, the thigh, and the buttocks, but none involved the vulva. In our review of the literature, we found two cases of solitary NLCS on the vulva and one classic form [5-7].

Various treatment modalities have been reported for NLCS [8], and it is noteworthy that aside from our case there is another instance of recurrence following CO_2_ laser treatment [9]. Considering the challenges in daily activities such as walking and movement caused by the large size and difficult locations of the lesions [10,11], we took into account firstly the functional aspects and secondly the aesthetic considerations. Consequently, we combined two procedures described in the literature for large NLCS lesions in challenging locations. We performed excisional surgery of the biggest mass, electrodesiccation of the multiple minor nodules, and healing by secondary intention, applying mupirocin cream twice a day for 10 days.

## Conclusions

Giant NLCS on the vulva is an extremely rare presentation. Successful treatment involves a combination of surgical excision and electrodessication, considering functional and aesthetic considerations. Further research and reported cases are needed to enhance our understanding of this rare condition and guide optimal treatment strategies.

## References

[1] Baraldi C, Barisani A, Fanti PA, Patrizi A (2021). Clinical, dermoscopic and histopathological features of solitary nevus lipomatosus cutaneous superficialis. Indian J Dermatol Venereol Leprol.

[2] Li S, Xiao Y, Wang H, Liu Z (2022). Giant nevus lipomatosus cutaneous superficialis with cerebriform surfaces on the back and sacral region: a case report. Clin Cosmet Investig Dermatol.

[3] Goucha S, Khaled A, Zéglaoui F, Rammeh S, Zermani R, Fazaa B (2011). Nevus lipomatosus cutaneous superficialis: report of eight cases. Dermatol Ther (Heidelb).

[4] Alotaibi H, Alsaif F, Alali A, Almashali M, Al-Dabeeb D, Altaweel AA (2018). Nevus lipomatosis cutaneous superficialis: a single-center case series of 5 patients. Case Rep Dermatol.

[5] Nakashima K, Yoshida Y, Yamamoto O (2010). Nevus lipomatosus cutaneous superficialis of the vulva. Eur J Dermatol.

[6] Hattori R, Kubo T, Yano K, Tanemura A, Yamaguchi Y, Itami S, Hosokawa K (2003). Nevus lipomatosus cutaneous superficialis of the clitoris. Dermatol Surg.

[7] Singh N, Kumari R, Thappa DM, Kar R, Kulandaisamy S (2014). Classic form of nevus lipomatosis cutaneous superficialis of vulva. Indian J Dermatol Venereol Leprol.

[8] Fatah S, Ellis R, Seukeran DC, Carmichael AJ (2010). Successful CO2 laser treatment of naevus lipomatosus cutaneous superficialis. Clin Exp Dermatol.

[9] Kim YJ, Choi JH, Kim H, Nam SH, Choi YW (2012). Recurrence of nevus lipomatosus cutaneous superficialis after CO2 laser treatment. Arch Plast Surg.

[10] Ancer-Arellano J, Villarreal-Villarreal CD, Cardenas-de la Garza JA, Cuellar-Barboza A, Vazquez-Martínez O, Ocampo-Candiani J (2019). Electrodissection for nevus lipomatosus cutaneous superficialis removal. J Am Acad Dermatol.

[11] Lu X, Zhang Q, Bu W, Fang F (2021). Giant naevus lipomatosus cutaneous superficialis on the buttock. Postepy Dermatol Alergol.

